# Relating Neuroticism to Emotional Exhaustion: A Dynamic Approach to Personality

**DOI:** 10.3389/fpsyg.2019.02264

**Published:** 2019-10-16

**Authors:** Joanna Sosnowska, Filip De Fruyt, Joeri Hofmans

**Affiliations:** ^1^Work and Organizational Psychology Group, Department of Psychology, Vrije Universiteit Brussel, Brussels, Belgium; ^2^Department of Developmental, Personality and Social Psychology, Ghent University, Ghent, Belgium

**Keywords:** personality, dynamics, burnout, emotional exhaustion, neuroticism

## Abstract

We build on a novel model of personality [PersDyn] that captures three sources of individual differences (here applied to neuroticism): (1) one’s baseline level of behavior, affect, and cognitions (baseline); (2) the extent to which people experience different neuroticism levels (variability); and (3) the swiftness with which they return to their neuroticism baseline once they deviated from it (attractor strength). To illustrate the model, we apply the PersDyn model to the study of the relationship between neuroticism and emotional exhaustion. In the first study, we conducted a 5-day experience sampling study on 89 employees who reported on their level of state neuroticism six times per day. We found that higher levels of baseline neuroticism and variability were related to increased emotional exhaustion. Furthermore, we found an interaction effect between baseline and attractor strength: people with a high baseline and high attractor strength tend to experience a high degree of emotional exhaustion, whereas people with low levels of baseline neuroticism are less likely to suffer from exhaustion if their attractor strength is high. In the second study, we conducted a laboratory experiment on 163 participants, in which we manipulated state neuroticism *via* short movie clips. Although the PersDyn parameters were not related to post-experiment emotional exhaustion, the interaction effect between baseline and attractor strength was replicated. It is concluded that a dynamic approach to neuroticism is important in understanding emotional exhaustion.

## Introduction

Personality is often used by scientists and practitioners as a predictor of work-related attitudes and behaviors. This practice is supported by a vast amount of research showing that personality relates to a wide range of work-related outcomes. For example, meta-analytic research (e.g., Barrick and Mount, 1991; Barrick et al., 2001) has shown that personality adds incremental value above and beyond mental ability or bio-data when predicting work performance, and despite an ongoing debate on the strength of those relationships, there is by now fair agreement that personality does indeed matter in the workplace and that personality assessment constitutes a valid part of many selection and recruitment processes (Hogan and Holland, 2003; Hogan, 2004; Judge and Zapata, 2015).

Traditionally, studies on the effect of personality at work have strongly focused on the predictive role of personality traits. In these studies, stable, between-person differences in personality traits have been related to stable, between-person differences in work behaviors and attitudes. During the last years, however, realization has grown that people not only differ from each other regarding their predisposition to behave, think, and feel in a specific way, but that these acts, thoughts, and feelings also fluctuate substantially across situations and time within one individual. In response to this, a line of research has been developing relatively independently from the trait approach, with a strong focus on within-person fluctuations or personality states (Fleeson, 2001; Debusscher et al., 2014; Dalal et al., 2015; Hofmans et al., 2015; Judge and Zapata, 2015; Jones et al., 2017). This increased attention to within-person fluctuations characterizes other research fields as well. For example, in their systematic review, McCormick and colleagues (McCormick et al., in press) observed a “meteoric rise in the number of management studies focused on within-person phenomena” (p. 19). Similarly, Podsakoff et al. (2019) found that more than half of the studies on within-person fluctuations were published in the last 4 years.

Although the research streams on traits and states have undoubtedly contributed to a better understanding of personality and its consequences in an occupational context, researchers have started to realize that, if we really want to understand personality and its effects at work, it does not suffice to study traits and states in isolation. Instead, an integrative approach that combines both traits and states is needed (Judge et al., 2014; Baumert et al., 2017; Fleeson, 2017). In the present research, we provide such an integrative approach to personality by conceptualizing personality as a dynamic system that combines within-person fluctuations and between-person differences. In particular, we draw on the recently developed Personality Dynamics model (PersDyn; Sosnowska et al., 2019a), a model that captures individual differences in three components of one’s personality system: (1) one’s baseline level of behavior, affect, and cognitions (baseline personality); (2) the extent to which people vary around this baseline (personality variability); and (3) the swiftness with which they return to their baseline once they deviated from it [personality attractor strength]. Using the PersDyn model, we study the relationship between neuroticism and emotional exhaustion, showing how conceptualizing personality as a dynamic system can contribute to understanding how personality relates to wellbeing-related outcomes at work.

### The Personality Dynamics Model

Recently, the notion that personality is characterized by both trait-related stability and intra-individual variability has received increased research attention. According to this approach, traits and states jointly influence behavior, implying that both are fundamental to understanding personality and behavior (Judge et al., 2014; Baumert et al., 2017). Moreover, by showing that traits predict states, previous research has shown that there is a correspondence between the trait level and in-the-moment description of relevant traits (Fleeson and Gallagher, 2009; Ching et al., 2014; Debusscher et al., 2014; Judge et al., 2014; Hofmans et al., 2015; Huang and Bramble, 2016).

A popular approach to the integration of traits and states is the density distribution approach by Fleeson (2001). The density distribution approach draws on the idea that, although traits are useful in predicting behavior over longer periods of time, in their day-to-day behavior people actively display a wide range of trait levels (Fleeson and Noftle, 2008; Fleeson and Jayawickreme, 2015). The consequence of this is that, because traits are manifested in people’s volatile day-to-day behaviors, the average level of these behaviors does not capture the entire spectrum of the individual’s behaviors and is therefore an incomplete indicator of personality. As a solution, the density distribution approach proposes to not describe personality using only one’s average state level, but to describe one’s personality using one’s entire distribution of states.

Recently, Sosnowska et al. (2019a,b) extended the idea of the density distribution approach in their Personality Dynamics (PersDyn) model. Similar to the density distribution approach, the PersDyn model is based on the idea that personality is reflected in the way traits are manifested on a momentary basis. However, the PersDyn model extends the density distribution approach by not only modeling the extent to which people vary in their momentary trait manifestations, but by also modeling the timing along which these changes occur. To achieve this goal, the PersDyn model makes use of three elements: (1) one’s home base (i.e., a baseline attractor state around which one’s personality states fluctuate); (2) the amount of variability around this home base; and (3) the swiftness with which people return to their home base once they deviated from it.

In what follows, we use the PersDyn model to look at the relation between neuroticism and emotional exhaustion. In particular, we will first discuss emotional exhaustion, after which we will explain how individual differences in baseline neuroticism, neuroticism variability, and neuroticism attractor strength are expected to relate to individual differences in emotional exhaustion.

### Individual Differences in Emotional Exhaustion

Emotional exhaustion is one of the three components of burnout, with burnout representing an affective reaction and response to ongoing stress, causing deterioration of emotional and cognitive resources over time (Shirom, 2003). Emotional exhaustion, being the discharge of energy and the excessive consumption of emotional resources (Bakker et al., 2006), affects physical and mental health, our behavior and attitudes (e.g., Maslach et al., 2001), and links more strongly with important life and work outcomes than any of the other components of burnout (Lee and Ashforth, 1996; Wright and Bonnett, 1997; Swider and Zimmerman, 2010; Alarcon, 2011). Hence, several authors have argued that emotional exhaustion captures the “core meaning” of burnout (Pines and Aronson, 1983; Wright and Cropanzano, 1998; Cropanzano et al., 2003).

Research on burnout and emotional exhaustion has focused mostly on the consequences of emotional exhaustion, while less attention has been devoted to what makes people prone to experiencing burnout and high levels of emotional exhaustion. Studies that have looked at the antecedents of emotional exhaustion have demonstrated that situational factors, and particularly characteristics in the workplace environment, are predictive of individual differences in emotional exhaustion (Maslach et al., 2001). Yet, its prevalence may differ not only across situations but also across individuals, with research showing that, several personality characteristics predispose some employees to experience higher levels of burnout and emotional exhaustion than others (Swider and Zimmerman, 2010; Golonka et al., 2019). Focusing on such person-related characteristics as a predictor of emotional exhaustion is of crucial importance as it might help identifying those individuals who are more prone to burnout and exhaustion than others.

### Neuroticism and Emotional Exhaustion

There are several reasons why personality is expected to relate to emotional exhaustion. First, people tend to select situations that match their personality (Frederickx and Hofmans, 2014), implying that there is a relationship between personality and the choice for certain job types (Wille and De Fruyt, 2014). For example, people of the “feeling type” may be inclined to choose a career in nursing and therefore end up in a highly stressful occupation (Garden, 1989). Second, personality predisposes people to experience the same situation in a different way. For example, research has shown that neurotic people experience the same situation as more stressful than people scoring low on neuroticism (Bolger and Schilling, 1991). Finally, personality also influences one’s coping strategies, with for example people scoring high on conscientiousness using more efficient coping strategies than people low on conscientiousness (Wayne et al., 2004).

Of the Big Five personality dimensions, neuroticism is the dimension that has most often been linked to burnout and emotional exhaustion (e.g., Lingard, 2003; LePine et al., 2004; Zellars et al., 2004; Bianchi, 2018), with high levels of neuroticism having well-documented effects on the physical (Lahey, 2009), cognitive (Colbert et al., 2004), and emotional (Judge et al., 1999) facets of global burnout. Moreover, the characteristics of emotional stability are well aligned with the indicators of emotional exhaustion (Anvari et al., 2011), with neuroticism predicting exhaustion even when organizational, demographic, and job stressors are controlled for (Zellars et al., 2004). In what follows, we will argue that we expect emotional exhaustion to relate to all three elements of the PersDyn model (i.e., baseline, variability, and attractor strength).

#### Baseline Neuroticism and Emotional Exhaustion

The first PersDyn component is the baseline. The baseline is derived from a series of momentary states and represents the point around which our behaviors, thoughts, and emotions fluctuate over time. In other words, it is the state toward which the individual’s behaviors, feelings, and cognitions converge. Thus, the baseline plays an important role as a standard for self-regulation by providing a point of reference that allows maintaining the system’s stability, even when such self-regulation is negative (Vallacher and Nowak, 2007). For example, if someone has a high neuroticism baseline, in the presence of situational influences which trigger momentary fluctuations from this baseline (e.g., acting in a calm manner), the person will have the tendency to go back to this highly neurotic state after a certain amount of time.

The idea to characterize individuals by means of baseline personality is central to all trait assessments of personality (Costa and McCrae, 1992). A vast amount of research supports the idea that the individual’s mean level of state personality over time is meaningful (Hamaker et al., 2007). For example, Furr (2009) proposed the concept of a psychological profile, in which the profile’s elevation is conceptually similar to our baseline, denoting the general level of behavior aggregated across situations. A similar concept was proposed by Shoda et al. (2002): in their model, the home base is described as an attractor, a set of stable states toward which the system is drawn. Finally, in the density distribution approach (Fleeson, 2001), baseline is the location of the highest density of the individual’s states one of the key elements of one’s personality system.

There are several reasons why individual differences in baseline neuroticism are expected to relate to individual differences in emotional exhaustion. First, research has shown that trait neuroticism is associated with a tendency to view the world negatively and see the environment as threatening and therefore depleting of resources (Bolger and Schilling, 1991; McCrae and John, 1992; Schneider, 2004). Second, people high in trait neuroticism tend to select situations that are in line with their personality and therefore end up experiencing more stressful (Bolger and Schilling, 1991) and negative events (Magnus et al., 1993; Frederickx and Hofmans, 2014). Third, highly neurotic people are characterized by increased stress sensibility and they are therefore more susceptible to negative stimuli than people low on neuroticism, which may also explain the link with emotional exhaustion (Bolger and Schilling, 1991; Larsen, 1992; Suls, 2001). Fourth, neurotic people find it more difficult to cope with stressful events, and they tend to use ineffective coping strategies, such as avoiding and distracting, denying, self-criticism, wishful thinking, which is yet another important factor that leads to energy depletion (Heppner et al., 1995). Hence, previous research has already demonstrated that people with high levels of trait neuroticism display high levels of post-work exhaustion, regardless of their pre-work levels of exhaustion, while for those with low levels of neuroticism post-work exhaustion depends on their level of pre-work exhaustion (Kammeyer-Mueller et al., 2016). In line with these findings, we expect baseline neuroticism to positively relate to emotional exhaustion.

Hypothesis 1: People with a high level of baseline neuroticism will be more likely to experience high levels of emotional exhaustion.

#### Neuroticism Variability and Emotional Exhaustion

Whereas the baseline captures consistency in one’s personality states (as it represents the state to which the system is drawn), the second component—variability, or the extent to which the individual varies around the baseline—captures variation in the personality system. Because the situational forces that affect our personality states vary in strength and direction, the actual behaviors, feelings, and cognitions will vary between individuals, even in very similar situations (Fleeson, 2007). Relevant to this issue is that previous research has shown that there are stable, between-person differences in the extent to which people’s personality-related states fluctuate (Moskowitz and Zuroff, 2004), which is why variability is sometimes referred to as “consistency in inconsistency” (Roberts, 2009). In sum, all of this suggests that it is meaningful to characterize one’s personality system using the extent to which one’s behaviors, feelings, and cognitions vary over time.

There are several explanations why patterns of variability are stable over time and can be used to distinguish individuals (Orom and Cervone, 2009; Epskamp et al., 2016; Jones et al., 2017). First, people differ in their sensitivity to situational cues, which means that the same set of conditions can trigger completely different responses among individuals, depending on their perception of the situation (Fleeson, 2001; Sherman et al., 2015). Moreover, some people display higher discriminative facility, meaning that they are more likely to make informed, discriminative choices of coping strategies based on situational cues, which may in turn lead to more or less variability in their behaviors, cognitions, and feelings (i.e., Mischel and Shoda, 1995, 1998; Mischel, 1977). Another concept that plays a major role in how people react to situations is personality strength. Whereas a strong personality encourages similar behavior from the individual, regardless of the situation, a weak personality provides little behavioral guidelines (Dalal et al., 2015). As a result, people with a strong personality are characterized by low variability, while people with a weak personality show high behavioral variability.

To understand the relationship between neuroticism variability and emotional exhaustion, we draw from research on counterdispositional behavior. Research on the consequences of counterdispositional behavior suggests that acting outside of one’s typical range of behaviors requires more effort and self-control than acting close to one’s typical range of behaviors (Neal et al., 2006; Pickett et al., 2019a,b). The reason is that habitual, well-learnt behavioral patterns do not require attention and conscious thought, while executive control is needed to deviate from these habitual behaviors, which in turn drains individual’s resources and may lead to emotional exhaustion (Schmeichel, 2007). Moreover, not only the heightened levels of cognitive control are energy depleting, also the feelings of inauthenticity and psychological conflict triggered by such counterdispositional behaviors have a negative impact on the individual’s emotional state (McGregor et al., 2006).

Importantly, not all counterdispositional behaviors are alike, with behaviors that deviate more strongly from one’s typical level of behavior requiring more effort to enact and maintain than behaviors closer to one’s typical level of behavior (Gallagher et al., 2011). In the PersDyn model, such deviations are captured by the personality variability component. High levels of variability imply that the individual shows very different levels of state neuroticism across time, which is indicative of frequent and/or severe acts of counterdispositional behavior. Low levels of variability, in turn, denote that the personality states of the individual are always close to his/her baseline personality. Because moving away from the baseline is known to be exhausting (Gallagher et al., 2011), we expect higher levels of neuroticism variability to relate to higher levels of emotional exhaustion.

Hypothesis 2: Individuals with higher levels of neuroticism variability will be more likely to experience emotional exhaustion.

#### Neuroticism Attractor Strength and Emotional Exhaustion

The third and last element of the PersDyn model is attractor strength, representing the force that regulates the fluctuations around the home base. Attractor strength reflects how fast one is drawn back in the direction of the baseline once the person deviated from it. Because of this regulatory function, attractor strength captures the interplay between stability and change in the personality system.

The introduction of attractor strength as one of the key elements of the personality system is in line with the notion that people do not passively submit to what is happening to them but instead regulate their own behavior, thinking, and feelings (Baumeister et al., 2007). Moreover, because attractor strength reflects whether one wanders around after being pulled away from the baseline (i.e., low attractor strength) or returns to the baseline swiftly (i.e., high attractor strength), it is linked to coherence in personality (Nowak et al., 2005), allowing for general adaptation of the system (Fajkowska, 2015). That is, with a weak attractor strength, there is little self-regulation in the personality system and therefore the person’s behavior, feelings, and cognitions are at the mercy of external influences. This is not trivial as research suggests that there is a link between instability in the personality system (i.e., a weak attractor strength) and mental health issues such as bipolar depression or suicidality (Johnson and Nowak, 2002).

The effects of counterdispositional behavior have also been linked to self-regulation (Hoyle, 2006). The longer we act outside our usual range of behaviors—and thus fail to self-regulate our behavior—the more depleted our resources will get. The reason is that the effects of counterdispositional behavior grow stronger over time, and lead to even higher intra-individual variability because people are increasingly losing the resources that are required to self-regulate their behaviors, affects, and cognitions. Thus, individuals who return to their baseline level faster will be less likely to experience the negative costs of counterdispositional behavior. Instead, when people stay away from their baseline for a longer time, it will be more difficult to maintain the energy resources and avoid the negative effects of counterdispositional behavior, including heightened levels of emotional exhaustion. Since attractor strength represents the swiftness with which one is drawn back in the direction of the baseline once (s)he deviated from it, we expect neuroticism attractor strength to negatively relate to emotional exhaustion.

Hypothesis 3: People who have a weaker attractor strength will be more likely to experience emotional exhaustion.

## Study 1

### Method

#### Procedure

We conducted an experience sampling study in which participants were asked to report on their level of state neuroticism six times per day. In particular, using the Personal Analytics Companion (PACO) smartphone app, participants were asked to report on their momentary level of neuroticism at 9 am, 10 am, 11 am, 1 pm, 2 pm, and 3 pm. The experience sampling study ended when the participant participated for minimally five working days or when they responded to at least 25 signals. After completing the experience sampling study, participants received a link to an online questionnaire in which they reported on their level of emotional exhaustion.

#### Participants

In total, 106 Belgian employees—recruited by associates of the last author—took part in the experience sampling study. Of these 106 employees, 16 failed to complete the emotional exhaustion questionnaire. This resulted in a final sample of 90 people (49 women). Participants’ age varied between 22 and 55 years (mean age = 33, SD = 9.058), and they had an average of 9.5 years of working experience. Participants worked in different sectors, such as logistics, staffing, IT, and telecom. Participants were financially rewarded for their participation in the study (15 euros).

#### Materials

##### State Neuroticism

State neuroticism was assessed using the Mini Marker scale (Saucier, 1994), which is a short version of Goldberg’s unipolar Big Five personality traits markers (Goldberg, 1992). The Mini Marker scale measures neuroticism through eight adjectives (e.g. “jealous”), which are rated on 7-point Likert scale (ranging from 1 = extremely inaccurate to 7 = extremely accurate). Because we measured momentary expressions of neuroticism, we asked the participants to indicate to what extent the adjectives applied to them at that particular moment. As the state neuroticism measurements include both between-person and within-person variation, we calculated reliability of the scores using the multilevel confirmatory factor analysis approach by Geldhof et al. (2014). In this approach, the within-person factor model is separated from the between-person model and an omega reliability index is calculated for each level separately using the factor loadings and residuals at the relevant level. For state neuroticism, the within-person omega reliability was 0.80, while the between-person omega reliability equaled 0.87.

##### Emotional Exhaustion

Emotional exhaustion was measured using the UBOS-A burnout scale (Schaufeli and Van Dierendonck, 2001). The scale is a Dutch version of the Maslach Burnout Inventory – General Survey (MBI-GS; Maslach et al., 1986), and measures three sub-dimensions of burnout, including emotional exhaustion. The questionnaire includes five items for emotional exhaustion, which are rated on a 7-point Likert scale (e.g., “Working all day is really a strain to me”). Cronbach’s alpha reliability for the emotional exhaustion subscale equaled 0.85.

#### Analyses

Baseline neuroticism, neuroticism variability, and neuroticism attractor strength scores were obtained by modeling the experience sampling data using the Bayesian Hierarchical Ornstein-Uhlenbeck model (BHOUM; Kuppens et al., 2010; Oravecz et al., 2016). BHOUM is a multilevel process model describing individual differences in within-person fluctuations over time, available as an extension to Matlab or a standalone program. The model is based on stochastic differential equations and can be expressed using the following two equations:

$$Y_{p}\left(t\right)=\Theta_{p}\left(t\right)+\varepsilon_{p}\left(t\right)\;\left(\mathrm{measurement}\;\mathrm{equation}\right)$$

$$d\Theta_{p}\left(t\right)/dt=\beta_{p}\times \left(\mu_{p}-\Theta_{p}\left[t\right]\right)+\xi_{p}\left(t\right)\;\left(\mathrm{transition}\;\mathrm{equation}\right)$$

The measurement equation relates the observed state neuroticism scores to the true state neuroticism scores. To do so, it splits the observed score *Y_p_* (*t*) for person *p* a time *t* into *Θ_p_* (*t*), being the latent (or true) neuroticism level for person *p* at time point *t* and *ε_p_* (*t*), the measurement error for person *p* at time point *t*. The transition equation, in turn, describes the dynamics in the latent neuroticism level across time. In this equation, d*Θ_p_* (*t*) / d*t* represents the change in the latent neuroticism level at for person *p* at time point *t*, with these changes being a function of (1) the distance between the current neuroticism level *Θ_p_* [*t*] and person *p*’s neuroticism baseline *μ_p_*, (2) person *p*’s neuroticism attractor strength *β_p_*, and (3) a stochastic component *ξ_p_* (*t*), which adds random variation (or noise) to the system.

The deterministic part of the transition equation [i.e., *β_p_* × (*μ_p_* − *Θ_p_* [*t*])] shows how baseline, variability, and attractor strength are responsible for intra-individual fluctuations in neuroticism. If the current level of neuroticism is below the baseline [i.e., (*μ_p_* − *Θ_p_* [*t*]) > 0], the derivative becomes positive, which means that the predicted change in the level of neuroticism at time point t will be positive (in other words, the neuroticism level will increase). If the current level of neuroticism is above the baseline [i.e., (*μ_p_* − *Θ_p_* [*t*]) < 0], the derivative is negative and therefore the level of neuroticism is predicted to decrease. This clearly reflects the idea that the process is continuously pulled toward the baseline. Moreover, this process is affected by attractor strength *β_p_* in the sense that when *β_p_* is large, return to the baseline will occur faster. If attractor strength *β_p_* is small, the change toward the baseline level will occur at a slower rate.

In the BHOUM, the model parameters—which directly correspond to the elements of the PersDyn framework—are person-specific and therefore are allowed to vary between individuals (hence the subscript *p* in the equations). These random effects create the hierarchical structure suitable to examine inter-individual differences in baseline personality, personality variability, and personality attractor strength. To avoid computationally prohibitive integration of numerous random effects’ distributions, the BHOUM uses Bayesian analysis.

In the present paper, BHOUM was used to obtain the three person-specific parameters of the PersDyn model: neuroticism baseline, neuroticism variability, and neuroticism attractor strength. In the second step of the analysis, these person-specific parameters were related to people’s emotional exhaustion scores. Missing data in the repeated measurements of neuroticism are handled in a straightforward way, as the model is based on continuous time measurements. This means that, even though the neuroticism measurements were only taken at discrete time points, BHOUM assumes that the process unfolds continuously between these discrete measurement moments.

### Results

The dynamics in state neuroticism of the 106 people who participated in the experience sampling study (*N* = 3,207 unique observations) were modeled using a one-dimensional BHOUM. We used the default BHOUM settings for the sampling algorithm, meaning that Markov chain sampling was based on six chains (each starting from different starting values), with each chain consisting of 10,000 iterations. Burn-in, or the number of initial iterations that was discarded from the posterior distribution, was set to 4,000.

#### Individual Differences in Baseline, Variability, and Attractor Strength

Table 1 shows a summary of the BHOUM results for state neuroticism in terms of the posterior mean and the lower and upper limits for the symmetric 95% posterior credibility intervals (PCIs). On average, the baseline is 2.22 (posterior *M*), on a measurement scale ranging from 1 to 7, which indicates that the average level of baseline neuroticism in our sample is rather low. Of course, the baseline differed between individuals, with the inter-individual variation in baseline neuroticism being 0.35 (posterior *M*).

**Table 1:** State neuroticism modeled in BHOUM: summary of the results.

Model parameter	Posterior mean	95% posterior credibility interval	
Baseline	2.22	2.10	2.35
Inter-individual variation in baseline	0.35	0.25	0.48
Intra-individual variation	0.47	0.36	0.63
Inter-individual variation in intra-individual variation	0.50	0.17	1.33
Attractor strength	0.82	0.50	1.40
Inter-individual variation in attractor strength	6.21	0.63	30.11
Measurement error	0.04	0.04	0.05

The second BHOUM parameter is the amount of intra-individual variation. Results indicated that the differences in the level of neuroticism over time within an individual (posterior *M* = 0.47) are larger than the differences in baseline between individuals (posterior *M* = 0.35). In other words, there is more variation in neuroticism within individuals than there is variation between individuals. Furthermore, comparing the amount of intra-individual variation (posterior *M* = 0.47) and the amount of measurement error (posterior *M* = 0.04) shows that meaningful changes in state neuroticism exceed vastly the level of measurement error, with the latent process accounting for the largest part of variation in the data. Finally, and of particular importance for the PersDyn model, there appear to be substantial between-person differences in the extent to which people’s neuroticism levels vary over the course of the experience sampling study, which is shown by substantial between-person variation in the within-person variances (posterior *M* = 0.50).

Finally, the average centralizing tendency—represented by attractor strength—is 0.81(posterior *M*), and also for this PersDyn parameter, there are large individual differences, meaning that people differ substantially in the swiftness with which they return to their baseline level of neuroticism when having deviated from it (posterior *M* = 6.21).

#### Relating Individual Differences in Baseline, Variability, and Attractor Strength to Emotional Exhaustion

Having demonstrated that there are important between-person differences in baseline neuroticism, neuroticism variability, and neuroticism attractor strength, in the second step of the analysis, we focused on relating those between-person differences in each of the PersDyn components (i.e., baseline, variability, and attractor strength) to between-person differences in emotional exhaustion. Table 2 presents the correlation coefficients between all PersDyn components and emotional exhaustion. Among the PersDyn components, only baseline and variability were significantly correlated (*r* = 0.47, *p* < 0.01), implying that people with a higher neuroticism baseline also showed more variability in their level of state neuroticism. Attractor strength was not significantly related to baseline (*r* = −0.03, ns) or variability (*r* = 0.13, ns).

**Table 2:** Pearson’s correlations for PersDyn elements and emotional exhaustion.

		Emotional exhaustion	State neuroticism	
			Baseline	Variability
State neuroticism	Baseline	0.33^**^		
	Variability	0.25^*^	0.47^**^	
	Attractor strength	0.02	−0.03	0.13

* p < 0.05

** *p < 0.01*.

Linking the PersDyn components with emotional exhaustion showed that people with a higher level of baseline neuroticism suffered from a higher level of emotional exhaustion (*r* = 0.33; *p* < 0.01), which supports our first hypothesis. Similarly, and in line with Hypothesis 2, higher levels of neuroticism variability were related to increased levels of emotional exhaustion (*r* = 0.25; *p* < 0.05). Hypothesis 3, in turn, was not supported as attractor strength appeared to be unrelated to emotional exhaustion (*r* = 0.02, ns). When predicting emotional exhaustion based on the three PersDyn components simultaneously, baseline neuroticism (*β* = 0.56; *p* < 0.05), but not neuroticism variability (*β* = 0.28, ns), nor attractor strength (*β* = 0.02, ns), was a statistically significant predictor.

**Figure 1:**
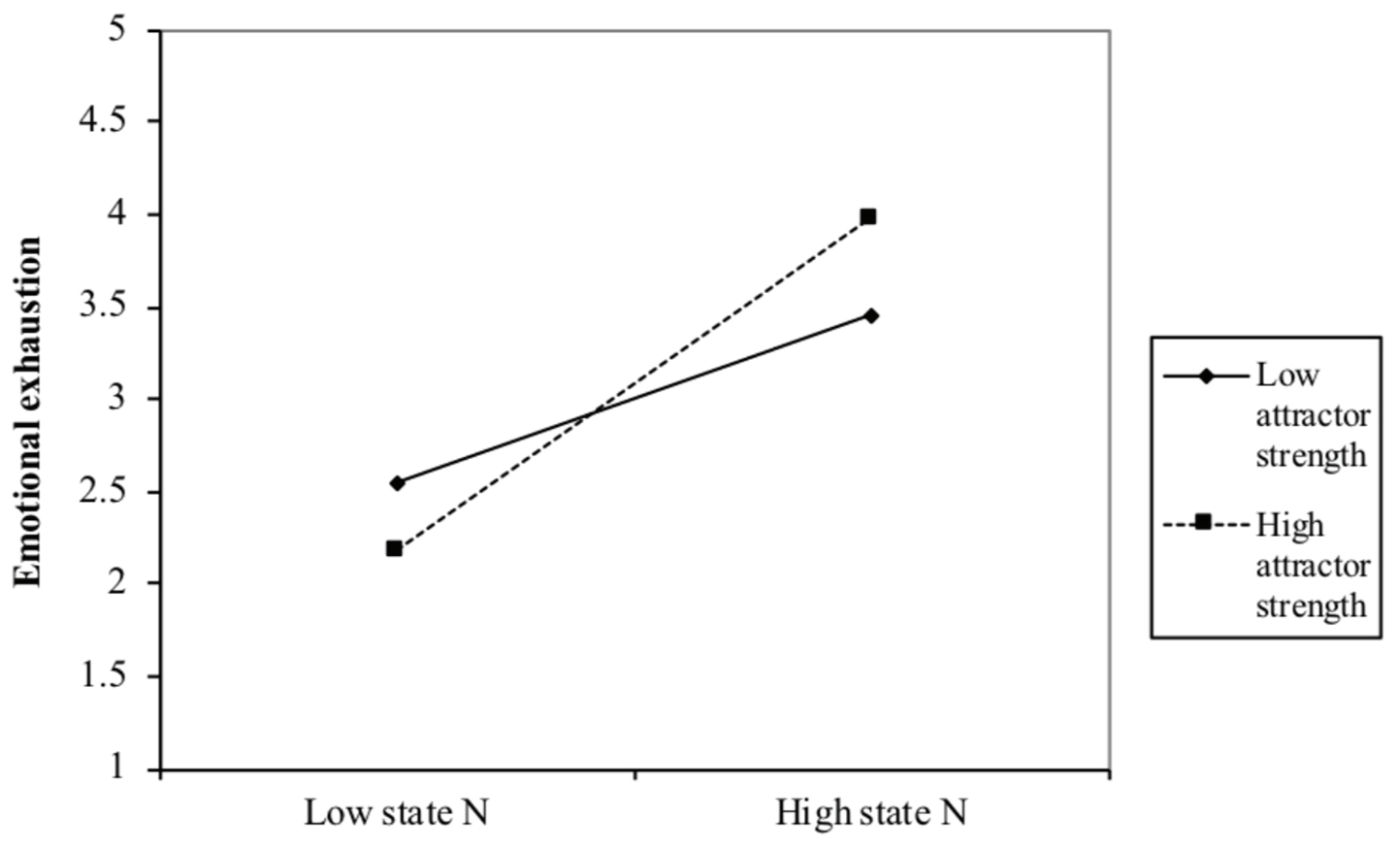
Interaction effect between neuroticism baseline and attractor strength in relation to emotional exhaustion.

Apart from the direct effects, exploratory follow-up analyses revealed an interaction effect between attractor strength and baseline neuroticism (*β* = 0.80; *p* < 0.05) (see Figure 1). To interpret this interaction effect, we performed a simple slopes analysis, which showed that people with a high attractor strength (+1 SD) experienced a higher degree of emotional exhaustion when their baseline was high than when it was low (*β* = 0.81; *p* < 0.01). For people with a low attractor strength (−1 SD), however, emotional exhaustion was unrelated to the level of baseline neuroticism (*β* = 0.27; ns). The Johnson-Neyman technique confirms this analysis, revealing that for 61.8% of the participants (the 61.8% highest attractor strength scores), the relationship between emotional exhaustion and baseline neuroticism was positive and statistically significant whereas for the other 38.2% the relationship was non-significant. Interestingly, these findings suggest that baseline neuroticism especially matters when one is pulled back to his/her baseline swiftly. If case attractor strength is low, between-person differences in baseline neuroticism appear to be unrelated to individual differences in emotional exhaustion.

### Discussion

A first important finding of this study is that it demonstrates that people not only differ in their average level of state neuroticism (i.e., baseline personality), but also in the extent to which their level of neuroticism varies across situations and time (i.e., personality variability) and in the extent to which they are pulled back to their neuroticism baseline after having deviated from it (i.e., personality attractor strength). This is an important finding because it shows that individual differences in how one generally behaves, feels, and thinks only capture part of one’s personality system.

Moreover, we demonstrated that the different PersDyn components matter by showing that individual differences in baseline neuroticism and neuroticism variability are positively related to individual differences in emotional exhaustion. Although neuroticism attractor strength was not directly related to emotional exhaustion, the interaction between neuroticism attractor strength and baseline neuroticism turned out to be significant. Although this interaction was not anticipated, it potentially has important implications because it implies that individual differences in baseline neuroticism only matter when people have the tendency to return swiftly to this baseline.

Despite these promising findings, the study suffers from two noteworthy limitations. First, because of the nature of experience sampling studies in general and our experience sampling study in particular, different participants might have been in very different situations during our study, and these different situations might partially account for our findings. For example, some people might have participated in our study in a period in which they experienced high levels of workload, while others might have experienced low levels of workload during the study. As workload has been shown to trigger within-person fluctuations in state neuroticism (Debusscher et al., 2016), individual differences in baseline neuroticism, neuroticism variability, and neuroticism attractor strength might (partly) reflect individual differences in the situations people are confronted with. Second, because the PersDyn model takes into account the temporal dynamics of the personality states, it might be sensitive to the timeframe of the study. In line with most studies on the density distribution approach (Fleeson, 2001), we chose to perform an experience sampling study that spanned five working days. However, it remains an open question whether minute-to-minute fluctuations in personality states can be characterized by the same process model, including baseline, variability, and attractor strength.

To address both issues, we performed a second—experimental—study with a large sample of undergraduates. Because in a lab experiment all participants are presented with identical situations, such a design allows studying whether people differ in their reaction to the same situational features.

## Study 2

### Method

#### Procedure

For our second study, we conducted an experiment. In terms of procedure, participants were invited to the laboratory and upon arrival, they signed an informed consent after which they were asked to fill out a questionnaire concerning their momentary level of emotional exhaustion. After completing the informed consent and the emotional exhaustion scale, participants were put in a cubicle and watched a series of short movies, used to manipulate state neuroticism.

Before starting the actual experiment, participants went through a practice trial in which they were presented with a short film clip (“The present,” 4 min). The aim of the practice trial was to get participants acquainted with the setup of the study. The first movie of the actual experiment (“Short term 12,” 21 min) is an emotionally intense movie, concerning a group home for troubled adolescents. The second movie (“The most relaxing video in the world,” 6 min) contains relaxing music and scenes from nature, such as a sunset, a sea view, and a forest. The third movie (“ReMoved,” 12 min) was again emotionally intense and was about a 9-year-old girl going through foster care system. The movies were cut into smaller scenes, with each scene representing a cohesive part of the story line. Although the first and last movies were used to elicit elevated levels of state neuroticism, the second movie allowed participants to recover and return to their baseline^1^.

During the experiment, the movies were paused at predefined times, each time asking participants to report on their level of state neuroticism using a slider that automatically popped up once the movie got paused. Using this procedure, participants reported on their level of state neuroticism 31 times in total. After seeing the movies, participants were asked once more to rate their momentary level of emotional exhaustion.

#### Participants

Participants were 163 undergraduate psychology students from a Western European university. On average, they were 19 years old (SD = 1.39), and 76% of participants were women (*n* = 124). Participation in the experiment was voluntary, and those who took part received one credit point for an Introductory Psychology course.

#### Materials

##### Emotional Exhaustion

An emotional exhaustion measure specifically designed for measuring emotional exhaustion in student populations was used (Schaufeli and Bakker, unpublished). Because participants had to rate their momentary level of emotional exhaustion, we made adjustments to the instructions and asked participants to rate their momentary level of emotional exhaustion, as opposed to the general level measured in Study 1. The Cronbach’s alpha reliability coefficient of the 5-item scale was 0.82 for the pre-experiment measure and 0.87 for the post-experiment measure.

##### State Neuroticism

State neuroticism was measured using a one-item semantic differential scale (Gosling et al., 2003), with one end of the scale representing adjectives describing a low level of state neuroticism (“calm, emotionally stable”) and the other end representing high levels of state neuroticism (“anxious, easily upset”)^2^. People rated this item using a slider with 21 possible scores.

### Results

As in the first study, we modeled the repeated measures data of the 163 participants (*N* = 5,509 unique observations) using the one-dimensional BHOUM. Again, the default BHOUM settings were used: six Markov sampling chains; 10,000 iterations per chain; and a burn-in of 4,000 iterations.

#### Individual Differences in Baseline, Variability, and Attractor Strength

The BHOUM results for state neuroticism are shown in Table 3, including the posterior mean as well as the 95% PCIs. The mean baseline was 8.36 (posterior *M*) on a scale from 0 to 20, indicating a relatively low average level of state neuroticism in the sample. Again, there was substantial inter-individual variation in baseline neuroticism (posterior *M* = 4.85). The average amount of within-person variation was 30.19 (posterior *M*), indicating that participant’s level of neuroticism varied more across the different measurements than that the participants differed from each other in their average level of neuroticism. Furthermore, participants substantially differed from each other in the extent to which they showed within-person variability, which can be seen from the large inter-individual variation in intra-individual variation (posterior *M* = 249.2). Finally, the average attractor strength was 21.92 (posterior *M*), with large between-person differences in this regulatory force (posterior *M* = 462.7)^3^.

**Table 3:** State neuroticism modeled in BHOUM: summary of the results of the lab experiment.

Model parameter	Posterior mean	95% posterior credibility interval	
Baseline	8.36	7.81	8.89
Inter-individual variation in baseline	4.85	2.32	7.93
Intra-individual variation	30.19	26.37	33.96
Inter-individual variation in intra-individual variation	249.2	73.3	578.7
Attractor strength	21.92	18.25	26.33
Inter-individual variation in attractor strength	462.7	161.3	993.5
Measurement error	0.05	0.01	0.15

#### Relating Individual Differences in Baseline, Variability, and Attractor Strength to Emotional Exhaustion

Table 4 contains the correlation coefficients between baseline neuroticism, neuroticism variability, neuroticism attractor strength, and the emotional exhaustion scores before and after the experiment. Among the PersDyn elements, the baseline was positively related to variability (*r* = 0.38, *p* < 0.01), and negatively to attractor strength (*r* = −0.20, *p* < 0.05). Variability and attractor strength were not significantly related (*r* = −0.14, ns). The PersDyn elements were not significantly correlated with the pre- and post-measure of emotional exhaustion.

**Table 4:** Pearson’s correlations for PersDyn elements and emotional exhaustion.

		Emotional exhaustion pre	Emotional exhaustion post	State neuroticism	
				Baseline	Variability
State neuroticism
	Emotional exhaustion post	0.86^**^			
	Baseline	0.02	0.00		
	Variability	0.02	−0.01	0.38^**^	
	Attractor strength	0.12	0.11	−0.20^*^	−0.14

* p < 0.05

** *p < 0.01*.

**Figure 2:**
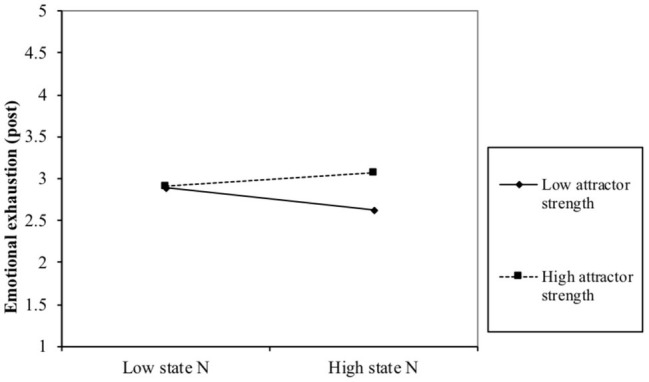
Interaction effect between neuroticism baseline and attractor strength in relation to emotional exhaustion after the experiment controlling for emotional exhaustion before the experiment.

Subsequently, we tested whether the PersDyn elements predicted post-experimental emotional exhaustion when controlling for pre-experimental emotional exhaustion. Partial correlation coefficients showed that baseline neuroticism (*r* = −0.01, ns), neuroticism variability (*r* = −0.05, ns), and neuroticism attractor strength (*r* = 0.01, ns) were unrelated to post-experimental emotional exhaustion. Similar to Study 1, we also tested interactions between the PersDyn elements, revealing that the interaction between neuroticism attractor strength and baseline neuroticism significantly predicted post-experimental emotional exhaustion when controlling for pre-experimental emotional exhaustion (*β* = 0.002; *p* < 0.05). To interpret the interaction, we performed a simple slope analysis (see Figure 2). This analysis revealed that for both people scoring 1 SD below (*β* = −0.07, ns) and 1 SD above the average attractor strength, the baseline level of neuroticism was unrelated to post-experimental emotional exhaustion (*β* = 0.05, ns). Subsequently, we performed a Johnson-Neyman analysis, which showed that for the 5% highest attractor strength scores the relationship between emotional exhaustion and baseline neuroticism was statistically significant and positive whereas for the other 95% the relationship was non-significant. Hence, this interaction pattern is in line with the finding of Study 1 that baseline neuroticism is only predictive of emotional exhaustion for people scoring (very) high on neuroticism attractor strength.

### Discussion

The results of our second study confirmed our earlier finding that people display a significant amount of within-person variability in their personality states, and that there are substantial between-person differences in the forces driving this within-person variability. Moreover, because all participants were presented with the same stimuli, between-person differences in the forces driving within-person neuroticism variability (i.e., baseline, variability, and attractor strength) cannot be explained by differential exposure to situations. Instead, the finding that people differ in baseline, variability, and attractor strength, even when presented with exactly the same situations, reveals that those individual differences are person-related and therefore capture important aspects of one’s personality system.

We also found an interaction between baseline neuroticism and neuroticism attractor strength, showing that, only when attractor strength is very high, individual differences in baseline neuroticism relate positively to the level of emotional exhaustion after the experiment. Thus, apart from showing the existence of individual differences in the PersDyn elements, we found some evidence that those PersDyn elements (jointly) predict relevant outcomes.

Despite some parallels between the findings of Study 2 and Study 1, there are also notable differences, such as the fact that baseline and variability were not directly related to emotional exhaustion. These differences might be due to various reasons. First, in our first study state neuroticism was measured over the time course of several days, whereas in the experimental study we studied minute-to-minute fluctuations in state neuroticism. Although we found substantial individual differences in all PersDyn model components in both studies, the correspondence between these individual differences remains an open question. In other words, it remains to be studied whether people can be characterized by the same PersDyn parameters when being observed on different timeframes. Second, it remains to be settled to what extent individual differences in baseline, variability, and attractor strength in a particular (and highly controlled) environment correspond with individual differences in baseline, variability, and attractor strength as measured in real life. For example, because of the emotionally intense nature of the movies, most people in the experimental study experienced elevated levels of state neuroticism, regardless of their general baseline neuroticism. As such, the baseline scores in the experimental study might only describe the participant’s baseline during the course of the experiment, and not their overall baseline neuroticism. For exactly this reason, we did not measure individual differences in the extent to which one generally feels emotionally exhausted, but individual differences in the level of emotional exhaustion at the end of the experiment. Whereas this makes sense from a substantive point of view, it introduces an additional difference with the first study (i.e., the scale in the second study was adjusted to measure momentary level of exhaustion). Finally, changing the neuroticism scale to a one-item differential scale introduced yet another difference in the study design, which may have impacted the results. Because of these differences, full correspondence between the results of both studies can probably not be expected. However, the fact that we found individual differences in baseline, variability, and attractor strength in a highly controlled experimental setting as well as in a real-life setting strengthens our claim that the PersDyn parameters are useful in describing people’s personality system.

## General Discussion

Personality is often defined as individual differences in stable ways of acting, thinking, and feeling (McCrae and Costa, 1999; Ashton and Lee, 2001; Barrick et al., 2001; Judge et al., 2008). In line with this definition, the traditional way of looking at personality is to focus on how we behave, think, and feel across a wide range of situations and contexts. Whereas such a static approach to personality undoubtedly served applied psychologists primarily interested in predictive validities, it fails to tap into the dynamic processes underlying personality functioning at work. In response to this, the personality literature has witnessed an increased attention for aspects of change, including research on short-term fluctuations (Fleeson and Gallagher, 2009) as well as long-term changes (Roberts et al., 2008). In the present paper, we used a model of personality that integrates change and stability by not only focusing on individual differences in the baseline level of personality, but also on individual differences in personality variability (the extent to which people differ in their personality states) and individual differences in attractor strength (the time it takes to return to his/her baseline). Notably, such a conceptualization of personality meets the three criteria that according to McCormick et al. (in press) optimize the contribution of within-person research: (1) the model explicitly includes temporality, (2) it elucidates within-person change over time, and (3) it yields findings that cannot be obtained using between-person research.

Using both high-density repeated measures data and experimental data on neuroticism, we empirically demonstrated that people indeed differ not only in their baseline level of neuroticism, but that there are also significant differences in the amount of intra-individual variation of neuroticism, and the swiftness with which they return to their baseline. Furthermore, we showed that these individual differences are instrumental in the prediction of individual differences in emotional exhaustion. By doing so, the present study offers a comprehensive perspective on personality that has the potential to contribute to advancing not only our fundamental understanding of personality but that also has the potential to contribute to applied personality research aimed at predicting work-related outcomes.

A unique feature of the PersDyn model, and one that sets it apart from other personality models, is that it links personality stability with change. By looking at personality as a dynamic system, the PersDyn model explicitly recognizes the fact that people affect the situations they are in as much as they are affected by these situations, and this bidirectionality shows in the notion of attractors. In the PersDyn model, the baseline represents the attractor state to which the system evolves over time and to which it returns when being perturbed (Nowak et al., 2005). This baseline represents the comfort zone the individual likes to return to, and because of this reason, it can be conceived of as the standard for self-regulation and stability in personality in the sense that it keeps the system in balance, creating “an emergent coherence around the attractor” (Kuppens et al., 2010; p. 1044). The notion of attractors might help to understand how change and stability interplay in one’s personality. In the present paper, we demonstrated that, in everyday life (but not in an experimental context), individual differences in baseline neuroticism were meaningfully linked with individual differences in emotional exhaustion, suggesting that one’s baseline captures a key element of people’s personality.

We also demonstrated that the extent to which people vary in their neuroticism states characterizes people, and that individual differences in variability were moderately related to individual differences in baseline (see also Fleeson, 2007). An important observation in both studies was that there was more within-person neuroticism variability than between-person neuroticism variability, indicating that the neuroticism levels of an individual vary more across situations than the average neuroticism levels vary across people. This is particularly relevant because the BHOUM allows separating meaningful intra-individual variance from measurement error, implying that dismissing intra-individual fluctuations as a measurement error conceals important information about people’s personality. In line with the idea that variability taps into an important aspect of personality, we showed in our first study that in a real-life context individual differences in emotional exhaustion could be predicted from individual differences in the variability of neuroticism, with people who vary more being more susceptible to high levels of emotional exhaustion.

Finally, our results confirmed the importance of looking at individual differences in attractor strength. The attractor strength parameter in the PersDyn model captures self-regulation in the personality system in the sense that it represents the extent to which the return to one’s baseline is swift and effective once the system is perturbed. In both studies, our results revealed that, for the prediction of individual differences in emotional exhaustion, attractor strength interacted with baseline neuroticism. Those with a high neuroticism baseline were more likely to suffer from emotional exhaustion, but only if they returned to their baseline swiftly. This finding suggests that swift self-regulation is not always beneficial for individual’s mental health, and that this is particularly true when one’s neuroticism baseline is high. Moreover, we found that, when attractor strength is low, individual differences in baseline neuroticism were unrelated to individual differences in emotional exhaustion. The reason is that, if self-regulation in the personality system is low, the attractor (i.e., baseline neuroticism) is not a distinguishing feature of the personality system because in that case one’s behavior, cognitions, and feelings are influenced by the situation rather than by one’s inner dispositions. Thus, in case of low attractor strength, the status of the average level of trait, cognitions, and feelings as an attractor can be questioned because this average level no longer represents a state that actively governs homeostasis in the system. This finding again underscores the importance of going beyond average levels of behaviors, cognitions, and feelings when describing personality.

## Limitations and Future Research Directions

In this paper, we applied the PersDyn model to the study emotional exhaustion using two different designs: an experience sampling method study and a laboratory experiment. We found some differences across the two studies: for example, although the interaction of baseline neuroticism and attractor strength was present in both studies, the strength of the interaction varied. The differences in the reported effect are potentially due to differences in the study design, such as the different length of data collection (2 weeks and 1 h) or the level of control over situational factors (high in the experimental design but low in the experience sampling study). Such differences should not come as a surprise, with Podsakoff et al. (2019) demonstrating that several method-related factors, such as the response format of the items and the time referent for the items, influence the findings of within-variability studies (more specifically the proportion of variance attributable to within-person differences). The consequence is that, because the results of our study cannot be fully evaluated without taking into account the differences in methodology, future research is necessary to further replicate our results across different settings and designs.

Next, it should be mentioned that the PersDyn model is likely to be trait specific in the sense that the effects of its elements and the interactions between the elements might differ depending on the trait under investigation. Therefore, further research is needed on the dynamics of other personality dimensions and on the effects of these dynamics.

Finally, whereas this paper focused on short-term changes in personality, the concept of personality dynamics can be also applied to long-term changes in personality, such as personality development (e.g., the TESSERA Framework, Wrzus and Roberts, 2017). Drawing on the idea that short-term changes can in the long term lead to long-term changes, future studies can use measurement burst design (e.g., week-long experience sampling study repeated every 3 months over several years) to examine whether the PersDyn model might advance our understanding of long-term personality changes.

## Conclusion

In the present study, we draw on the Personality Dynamics (PersDyn) model, a novel theoretical framework that captures individual differences in the dynamics of personality. Similar to the density distribution approach, the PersDyn builds on the idea that personality is reflected in the way traits are manifested on a momentary basis. At the same time, it extends previous approaches by not only modeling the extent to which people vary in their momentary trait manifestations, but by also modeling the timing along which these changes occur using three components: (1) one’s baseline level of behavior, affect, and cognitions (baseline); (2) the extent to which people fluctuate around this baseline (variability); and (3) the swiftness with which they return to their baseline once they deviated from it (attractor strength). To illustrate the usefulness of our model, we applied the PersDyn model to the study of the relationship between neuroticism and emotional exhaustion, showing that individual differences in the PersDyn parameters relate in a meaningful way to individual differences in emotional exhaustion.

## Nomenclature

### Resource Identification Initiative

SPSS, RRID:SCR_002865

MATLAB, RRID:SCR_001622

## Data Availability Statement

The datasets for this study can be found in the Open Science Framework https://osf.io/fnxt7/?view_only=eb6810df387f4ea087f2660d37bbf8e5.

## Ethics Statement

The ethical committee that approved this research is the Ethical Committee for Social Sciences and Humanities, Vrije Universiteit Brussel, Belgium.

## Author Contributions

JS, FF, and JH made substantial contributions to the conception and design of this paper, the acquisition, analysis, and interpretation of data, and drafting the manuscript and revising it critically for important intellectual content.

### Conflict of Interest

The authors declare that the research was conducted in the absence of any commercial or financial relationships that could be construed as a potential conflict of interest.

## Footnotes

^1^This idea was confirmed by a pilot study in which we ran two focus groups to evaluate whether each clip elicited changes in state neuroticism. Sixteen people participated in the focus groups. During each session, people were first shown a movie, after which they were asked to fill out a questionnaire in which they rated the extent to which they experienced changes in trait-relevant characteristics, such as “quarrelsome,” “anxious,” and “enthusiastic.” Using the Ten Item Personality Inventory (TIPI; Gosling et al., 2003), each of the Big Five traits was measured by two adjectives. Subsequently, we ran an open discussion, first explaining the purpose of the study and then asking participants if they considered the movies as a suitable way to manipulate the level of state neuroticism. The outcome from both the questionnaires and the open discussions suggested that the movies indeed induced changes in momentary levels of neuroticism, with the changes in neuroticism being larger than the changes in the other Big Five dimensions.

^2^In Gosling et al. (2003), the one item measure of neuroticism was “Emotionally stable, calm (that is, relaxed, self-confident, NOT anxious, moody, easily upset, or easily stressed).” In our study, we transformed this item into a semantic differential scale (instead of using brackets to indicate the opposite of low neuroticism) to ensure that the instructions were straightforward and clear.

^3^One might note that the attractor strength estimates are much larger than those in Study 1. It must be pointed out though that this metric is difficult to interpret in an absolute sense: while inter-individual differences in attractor strength are meaningful, the absolute metric itself is not and depends on several different factors, such as timing of the measurement and the rating scale used (see Kuppens et al., 2010).
